# Isolation and Complete Genome Sequence Analysis of *Kosakonia cowanii* Pa82, a Novel Pathogen Causing Bacterial Wilt on Patchouli

**DOI:** 10.3389/fmicb.2021.818228

**Published:** 2022-01-14

**Authors:** Yong Zhang, Bangwei Wang, Qiao Li, Derui Huang, Yuyao Zhang, Guangwei Li, Hong He

**Affiliations:** School of Pharmaceutical Sciences, Guangzhou University of Chinese Medicine, Guangzhou, China

**Keywords:** *Pogostemon cablin* (patchouli), bacterial wilt, pathogen isolation, complete genome sequencing, *Kosakonia cowanii*, virulence-related genes, functional validation

## Abstract

*Pogostemon cablin* (patchouli), an important medicinal and aromatic plant, is widely used in traditional Chinese medicine as well as in perfume industry. Patchouli plants are susceptible to bacterial wilt disease, which causes significant economic losses by reduction in yield and quality of the plant products. However, few studies focus on the pathogens causing bacterial wilt on patchouli. In this study, strain Pa82 was isolated from diseased patchouli plants with typical bacterial wilt symptoms in Guangdong province, China, and was confirmed to be a highly virulent pathogen of patchouli bacterial wilt. Comparative sequence analysis of 16S rRNA gene showed that the strain was closely related to *Kosakonia* sp. CCTCC M2018092 (99.9% similarity) and *Kosakonia cowanii* Esp_Z (99.8% similarity). Moreover, phylogenetic tree based on 16S rRNA gene sequences showed that the strain was affiliated with genus *Kosakonia*. Further, the whole genome of strain Pa82 was sequenced, and the sequences were assembled and annotated. The complete genome of the strain consists of one chromosome and three plasmids. Average nucleotide identity (ANI) and phylogenetic analysis revealed that the strain belongs to *Kosakonia cowanii* (designated *Kosakonia cowanii* Pa82). Virulence-related genes of the strain involved in adherence, biofilm formation, endotoxin and other virulence factors were predicted. Among them, *vgrG* gene that encodes one of the type VI secretion system components was functionally validated as a virulence factor in *Kosakonia cowanii* Pa82 through construction of Tn*5* insertion mutants and identification of mutant defective in virulence.

## Introduction

*Pogostemon cablin* (Blanco) Benth. (patchouli), a herbaceous plant species belonging to family *Lamiaceae*, is well known for its medicinal and aromatic properties. Patchouli is native to tropical Southeast Asia and now widely cultivated in many tropical and subtropical countries, especially China, India, Philippines, Indonesia, Vietnam, Malaysia, and Thailand (Swamy and Sinniah, 2016). In traditional Chinese medicine, the dried aerial parts of patchouli are frequently used for the treatment of headache, common cold, fever, nausea, vomiting, diarrhea, and so forth (Chen et al., 2021). Recently, extensive studies have indicated that patchouli possesses diverse pharmacological activities, including antimicrobial, antioxidative, antitumor, sedative, and gastrointestinal protective activities (Hu et al., 2017; Kim et al., 2019; Xie et al., 2020). Moreover, patchouli is also an economically important aromatic plant producing essential oil, which is widely used in fragrance and cosmetic industries (Chen et al., 2013).

Patchouli plants are susceptible to bacterial wilt disease, which causes significant economic losses by reduction in yield and quality of the plant products. Bacterial wilt is a devastating soil-borne disease that is widely distributed in tropical, subtropical, and some temperate regions, affecting hundreds of plant species, particularly the crops of family *Solanaceae*. Typical symptoms of the disease are leaf wilt, browning of vascular tissues, and even collapsing of the plant. *Ralstonia solanacearum* is known to be the most prominent causal agent of bacterial wilt worldwide, infecting a wide range of hosts, including solanaceous and non-solanaceous plants (Peeters et al., 2013; Jiang et al., 2017). In recent years, other causal organisms such as members of genus *Enterobacter*, genus *Erwinia* and genus *Kosakonia* have been reported to cause similar symptoms of bacterial wilt (Wang et al., 2008; Zhu et al., 2010; Sanogo et al., 2011; Sarkar and Chaudhuri, 2015).

In this study, strain Pa82 was isolated from diseased patchouli plants with typical bacterial wilt symptoms in Guangdong province, China, and was confirmed to be a highly virulent pathogen of patchouli bacterial wilt. And then, the complete genome of strain Pa82 was sequenced, and the sequences were assembled and annotated. Moreover, the approach for functional validation of potential virulence-related genes in strain Pa82 was established through construction of Tn*5* insertion mutants and identification of mutants defective in virulence. In this paper, we presented a novel pathogen causing bacterial wilt on patchouli and unraveled its phylogeny and genetic basis.

## Materials and Methods

### Pathogen Isolation

Naturally infected patchouli plants with typical symptoms of bacterial wilt were collected from Guangdong province, China. Vascular tissues at the base of the stems from these diseased plants were rinsed with sterile water, and then cut into several fragments. The fragments were soaked in sterile water, and the suspension was diluted and streaked on the medium containing 0.005% 2, 3, 5-triphenyltetrazolium chloride (TTC) (Swanson et al., 2005). From the plates, bacterial colonies were picked based on their morphology and were subcultured on fresh TTC medium until pure isolates were obtained. The pure isolates were stored at −80°C in nutrient agar (NA) liquid medium containing 7% dimethyl sulfoxide (DMSO).

### Morphological Observation

The bacterial isolates were streaked on TTC medium, and were cultured at 30°C for 36 h. Colony morphology of the bacterial isolates was then observed. Bacterial cells from each isolate were suspended in 0.9% NaCl solution. Following the Gram stain, cell morphology was observed under fluorescence microscopy using 100 × oil-immersion objective lens (1,000 × total magnification) (Azabou et al., 2007).

### Pathogenicity Test

The pathogenicity test of the bacterial isolates was conducted by inoculating the bacteria to patchouli plants. Surface sterilized leaf and stem explants of patchouli were cultured on Murashige and Skoog (MS) medium to achieve shoot regeneration, seedling propagation and regenerated shoot rooting. Plantlets with well-developed shoots and roots were transplanted into pots containing sterile soil and grown in greenhouse for about 40 days. A single colony of each bacterial isolate on NA solid medium was picked and transferred to NA liquid medium. After 24 h incubation at 28°C with continuous shaking (200 rpm), the bacterial suspension was diluted to approximately 5 × 10^8^ cfu mL^–1^. The adjusted bacterial suspension was inoculated into roots of patchouli plants using the soaking method while control plants were mock-inoculated sterile water (Vailleau et al., 2007). The plants were grown under greenhouse conditions maintained at 28 ± 2°C with 85% relative humidity.

### 16S rRNA Gene Sequence Analysis

Strain Pa82, a highly virulent strain isolated from diseased patchouli plants, was used for further strain analysis. After incubation at 28°C for 24 h, bacterial cells of strain Pa82 in NA liquid medium were harvested by centrifugation at 4,000 rpm for 10 min. Genomic DNA was extracted from the bacterial cells using Biospin Bacteria Genomic DNA Extraction Kit. The 16S rRNA gene sequence of strain Pa82 was amplified by polymerase chain reaction (PCR) using the forward primer 27F (5′-AGAGTTTGATCCTGGCTCAG-3′) and reverse primer 1541R (5′-AAGGAGGTGATCCAGCCGCA-3′). PCR reaction was conducted in a total volume of 25 μL containing 1 μL of DNA template, 1 μL of each primer, 12.5 μL of Premix Ex *Taq* and 9.5 μL of double-distilled water. PCR protocol was as follows: 94°C for 2 min; followed by 35 cycles at 94°C for 30 s, 57°C for 30 s, 72°C for 2 min; and a final extension at 72°C for 10 min. The PCR product was sequenced, and the sequence was compared with 16S rRNA gene sequences in the GenBank database. A phylogenetic tree based on the 16S rRNA gene sequences from strain Pa82 and its relative species was constructed using neighbor-joining method in MEGA X software and bootstrap analysis was performed with 1,000 replicates (Russo and Selvatti, 2018).

### Genome Sequencing and Assembly

Genomic DNA of strain Pa82 was extracted using the Wizard Genomic DNA Purification Kit according to the manufacturer’s instructions. The quality and quantity of the genomic DNA was determined using TBS-380 fluorometer. Short-read libraries (2 × 150 bp) were sequenced on the Illumina HiSeq X Ten platform and long-read libraries (about 10 kb) were sequenced on the Pacific Bioscience (PacBio) Sequel platform. The high-quality PacBio clean reads were then used for genome assembly by using Canu (Koren et al., 2017). Meanwhile the Illumina clean reads were used to correct errors for further improve the accuracy of the PacBio assembly results. The final genome assembly comprised seamless chromosomes and plasmids.

### Genome Annotation and Average Nucleotide Identity Analysis

Coding sequences (CDS), ribosomal RNA (rRNA) genes, and transfer RNA (tRNA) genes were predicted using Glimmer 3.02, Barrnap 0.8, and tRNAscan-SE 2.0, respectively (Delcher et al., 2007; Seemann, 2013; Lowe and Chan, 2016). The circular genome map was drawn using CGView 2 (Stothard and Wishart, 2005). All predicted CDS were searched and annotated by BLAST against the publicly available protein databases including NCBI non-redundant protein (NR), Swiss-Prot, Pfam, and Clusters of Orthologous Groups (COG). Average nucleotide identity (ANI) values between genomes of strain Pa82 and its relative strains were calculated using JSpeciesWS (Richter et al., 2016). Potential virulence-related genes were predicted by the BLAST search against the virulence factor database (VFDB) (Chen et al., 2016).

### Construction and Analysis of Tn*5* Transposon Insertion Mutants

Functional validation of predicted virulence-related genes in strain Pa82 requires the generation of gene disruption mutants. Mutants of strain Pa82 were constructed by using an EZ-Tn*5*™ < KAN-2 > Tnp Transposome™ Kit. Competent cells of the strain were prepared and mixed with Tn*5* transposomes, then the mixture was electrically transformed at 2.5 kV, 25 μF and 400 Ω. Transformed cells were recovered in SOC liquid medium at 30°C for 1 h with 200 rpm shaking and then plated on NA solid medium supplemented with 10 μg mL^–1^ kanamycin. Positive clones were identified by PCR with the specific primer pairs Kan-F and Kan-R, which were designed according to the sequence of the kanamycin resistance gene (*Kan*^r^) of Tn*5*. From these mutants, the flanking sequences adjacent to Tn*5* transposon insertion-site were amplified by inverse PCR using transposon-specific primer pairs KAN-2 FP-1 and KAN-2 RP-1, and the PCR products were sequenced (Malamud et al., 2012). The sequences were then compared with corresponding sequences available in GenBank database using BLAST.

## Results

### Isolation and Characterization of Strain Pa82

**FIGURE 1:**
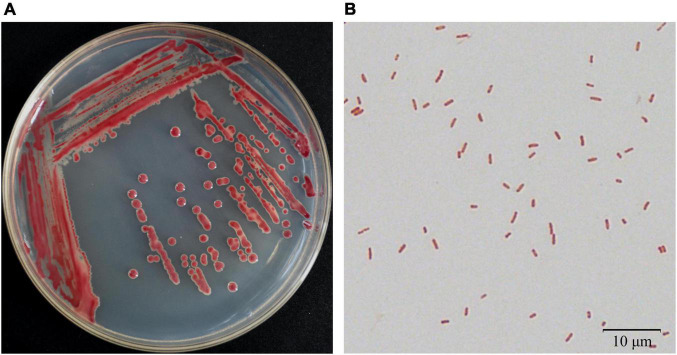
Morphological characteristics of strain Pa82. **(A)** Colony appearance on TTC medium; **(B)** Cell morphology under 100 × oil-immersion objective lens (1,000 × total magnification).

**FIGURE 2:**
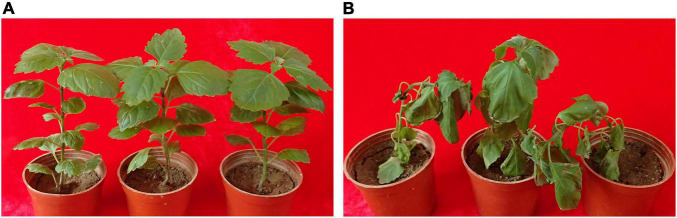
The plant phenotypes of pathogenicity test at 7 days post-inoculation. **(A)** Plants mock-inoculated with sterile water; **(B)** Plants inoculated with strain Pa82.

Bacteria were isolated from the patchouli plants infected with bacterial wilt by the streak plate method. The pathogenic isolates were evaluated by molecular, physiological and pathological characterization. Among them, strain Pa82 was found to be a highly virulent pathogen to cause patchouli bacterial wilt. The morphological characteristics of strain Pa82 are shown in Figure 1. The colonies are smooth, moist, circular disc with white edge and red pigmentation in the center when incubated on TTC medium at 30°C for 36 h (Figure 1A). The bacterial cells are Gram-negative and rod-shaped, ranging in length from 0.7 to 2.1 μm under the oil-immersion lens (Figure 1B). The virulence characteristics of strain Pa82 were investigated by conventional pathogenicity test. Patchouli plants inoculated with strain Pa82 developed initial symptoms as drooping and shriveling leaves, followed by complete wilting or collapsing of the whole plants, showing typical symptoms of bacterial wilt. Whereas control plants treated with sterile water did not exhibit disease-like symptoms and remained healthy. The plant phenotypes of pathogenicity test at 7 days post-inoculation are shown in Figure 2.

### 16S rRNA Phylogenetic Analysis of Strain Pa82

**FIGURE 3:**
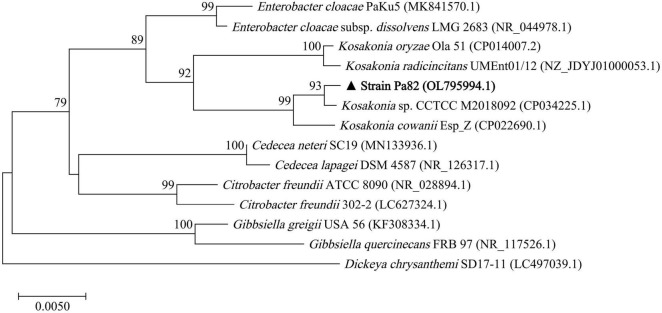
Phylogenetic tree based on 16S rRNA gene sequences from strain Pa82 and its relative strains of family *Enterobacteriaceae*. The 16S rRNA gene sequence from *Dickeya chrysanthemi* SD17-11 was used as the outgroup.

The 16S rRNA gene of strain Pa82 was amplified by PCR, and the sequencing data (1,460 bp in length) was submitted to NCBI database (accession number: OL795994.1). Comparative sequence analysis of 16S rRNA gene showed that the strain was closely related to *Kosakonia* sp. CCTCC M2018092 (99.9% similarity) and *Kosakonia cowanii* Esp_Z (99.8% similarity). As genus *Kosakonia* belongs to family *Enterobacteriaceae*, a phylogenetic tree based on the 16S rRNA gene sequences from strain Pa82 and its relative species belonging to family *Enterobacteriaceae* was constructed. The 16S rRNA gene sequence from *Dickeya chrysanthemi* SD17-11 was used as the outgroup. Horizontal branch lengths represent relative evolutionary distances. Numbers above branch nodes are bootstrap values. The phylogenetic tree showed that all the species were divided into three clades, and one of which included strain Pa82 and species from genus *Enterobacter* or genus *Kosakonia*. The results also showed that strain Pa82 was most closely related to *Kosakonia* sp. CCTCC M2018092, indicating that the strain was affiliated with genus *Kosakonia* (Figure 3).

### General Genome Features of Strain Pa82

**FIGURE 4:**
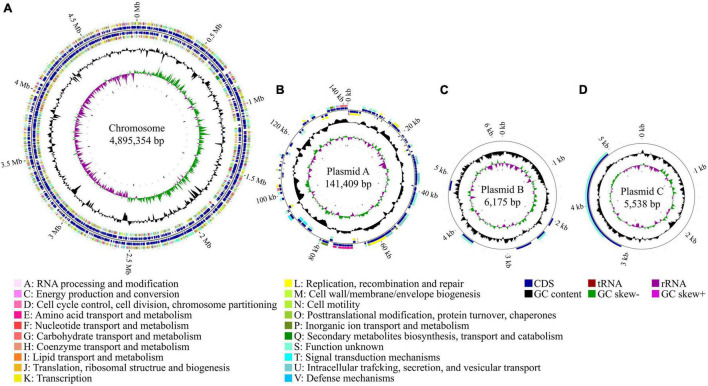
Circular maps of strain Pa82 genome. **(A)** Chromosome; **(B)** Plasmid A; **(C)** Plasmid B; **(D)** Plasmid C. The distribution of circles from inside to outside, circle 1, GC skew (green indicates a region with G content greater than C content; purple indicates a region with G content less than C content); circle 2, GC content (outward means above the average GC content of the whole genome; inward means below the average GC content of the whole genome); circles 3 and 6, CDS on the reverse and forward strands were annotated by Clusters of Orthologous Groups (COG) database (different colors represent different COG functional categories); circles 4 and 5, CDS, tRNA and rRNA on the reverse and forward strands; circle 7, scale marks of the genome size.

To better understand the genetic basis of the virulence properties of strain Pa82, the complete genome sequencing was performed, and the sequences were assembled and annotated. The complete genome of the strain was found to contain one chromosome of 4,895,354 bp and three plasmids of 141,409, 6,175, and 5,538 bp, respectively (Figure 4). The general genome features of strain Pa82 are summarized in Table 1. In total, 4,669 coding sequences (CDS) were predicted with 4,505 in the chromosome and 164 in the three plasmids. Among them, 4,284 CDS were assigned into the functional categories in Clusters of Orthologous Groups (COG) database (Table 2). The main categories are G (carbohydrate transport and metabolism; 8.15%), K (transcription; 8.15%), E (amino acid transport and metabolism; 7.82%), P (inorganic ion transport and metabolism; 6.63%), M (cell wall/membrane/envelope biogenesis; 5.86%) and C (energy production and conversion; 5.30%). However, the results also showed that a large number of CDS were assigned into COG category S (function unknown, 29.48%), which may be revealed by further functional studies.

**TABLE 1:** General genome features of strain Pa82.

Feature	Chromosome	Plasmid A	Plasmid B	Plasmid C
Size (bp)	4,895,354	141,409	6,175	5,538
GC content (%)	56.09	53.41	46.70	50.13
Coding sequences (CDS)	4,505	150	6	8
rRNAs	22	0	0	0
tRNAs	83	0	0	0
ncRNAs	10	0	0	0

**TABLE 2:** COG functional categories of strain Pa82 genome.

Category	Description	Gene number	Percentage (%)
A	RNA processing and modification	1	0.02
C	Energy production and conversion	227	5.30
D	Cell cycle control, cell division, chromosome partitioning	40	0.93
E	Amino acid transport and metabolism	335	7.82
F	Nucleotide transport and metabolism	85	1.98
G	Carbohydrate transport and metabolism	349	8.15
H	Coenzyme transport and metabolism	128	2.99
I	Lipid transport and metabolism	84	1.96
J	Translation, ribosomal structure and biogenesis	179	4.18
K	Transcription	349	8.15
L	Replication, recombination and repair	177	4.13
M	Cell wall/membrane/envelope biogenesis	251	5.86
N	Cell motility	51	1.19
O	Posttranslational modification, protein turnover, chaperones	143	3.34
P	Inorganic ion transport and metabolism	284	6.63
Q	Secondary metabolites biosynthesis, transport and catabolism	47	1.10
S	Function unknown	1,263	29.48
T	Signal transduction mechanisms	158	3.69
U	Intracellular trafficking, secretion, and vesicular transport	88	2.05
V	Defense mechanisms	45	1.05

### Average Nucleotide Identity Analysis for Strain Pa82

**FIGURE 5:**
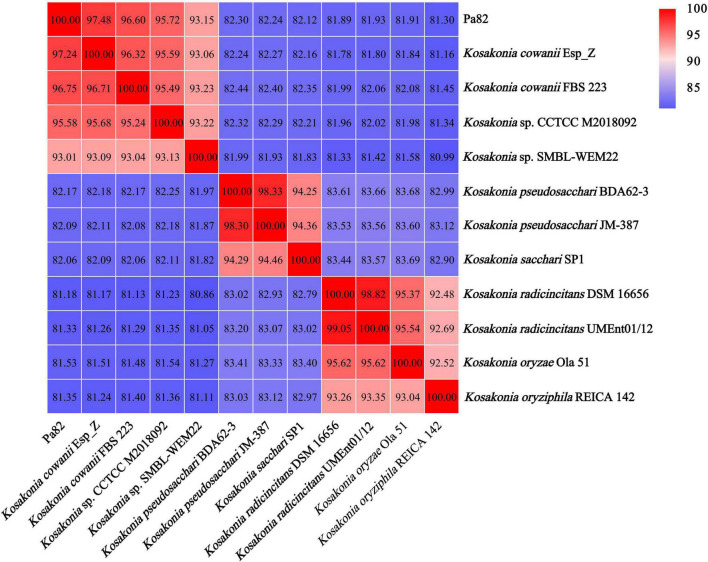
Heat map based on average nucleotide identity (ANI) values between each pair of genome sequences from strain Pa82 and strains within genus *Kosakonia*.

ANI was used to assess the relationship between strain Pa82 and its relative species from genus *Kosakonia*, and a threshold of 95–96% was set as a boundary for species delineation (Richter and Rosselló-Móra, 2009). The genome comparison between strain Pa82 and its relative species from genus *Kosakonia* showed ANI values ranging from 81.18 to 97.48% (Figure 5). Notably, the ANI value (97.48%) between the genome sequences of strain Pa82 and its closest related strain *Kosakonia cowanii* Esp_Z was above the accepted 95–96% cut-off threshold for species delineation. Therefore, combined with the ANI and phylogenetic analysis, strain Pa82 was identified as *Kosakonia cowanii* and designated as *Kosakonia cowanii* Pa82.

### Potential Virulence-Related Genes in Strain Pa82 Genome

Genes potentially involved in virulence of strain Pa82 were predicated using the VFDB. A total of 610 potential virulence-related genes were found in strain Pa82 genome with 601 in the chromosome and 9 in the plasmid A. Among them, genes involved in known and putative virulence factors which are important for bacterial infections are summarized in Table 3. These genes are classified into 6 categories, including adherence (9 genes), biofilm formation (3 genes), endotoxin (4 genes), invasion (23 genes), secretion system (6 genes), and toxin (2 genes).

**TABLE 3:** Potential virulence-related genes in strain Pa82 genome predicted by VFDB.

Virulence factor	Gene number	Gene name	Putative function
Adherence	9	*dcuS*	Two-component system sensor histidine kinase DcuS
		*fabG*	3-oxoacyl-ACP reductase FabG
		*fimA*	Type 1 fimbrial major subunit FimA
		*fimC*	Type 1 fimbria chaperone FimC
		*fimH*	Type 1 fimbrin D-mannose specific adhesin FimH
		*hisC*	Histidinol-phosphate transaminase
		*kduD*	2-dehydro-3-deoxy-D-gluconate 5-dehydrogenase KduD
		*uvrY*	UvrY/SirA/GacA family response regulator transcription factor
		*waaC*	Lipopolysaccharide heptosyltransferase RfaC
Biofilm formation	3	*cytR*	DNA-binding transcriptional regulator CytR
		*pdeH*	Cyclic-guanylate-specific phosphodiesterase
		*rbsR*	Ribose operon transcriptional repressor RbsR
Endotoxin	4	*arnB*	UDP-4-amino-4-deoxy-L-arabinose aminotransferase
		*galE*	UDP-glucose 4-epimerase GalE
		*galU*	UTP-glucose-1-phosphate uridylyltransferase GalU
		*rfbB*	dTDP-glucose 4,6-dehydratase
Invasion	23	*cheA*	Chemotaxis protein CheA
		*cheB*	Chemotaxis response regulator protein-glutamate methylesterase
		*cheW*	Chemotaxis protein CheW
		*cheY*	Chemotaxis response regulator CheY
		*flgD*	Flagellar hook assembly protein FlgD
		*flgE*	Flagellar hook protein FlgE
		*flgG*	Flagellar basal-body rod protein FlgG
		*flgK*	Flagellar hook-associated protein FlgK
		*flgN*	Flagella biosynthesis chaperone FlgN
		*flhA*	Formate hydrogenlyase transcriptional activator FlhA
		*flhC*	Flagellar transcriptional regulator FlhC
		*flhD*	Flagellar transcriptional regulator FlhD
		*fliA*	RNA polymerase sigma factor FliA
		*fliC*	Flagellin FliC
		*fliG*	Flagellar motor switch protein FliG
		*fliI*	Flagellum-specific ATP synthase FliI
		*fliM*	Flagellar motor switch protein FliM
		*fliP*	Flagellar type III secretion system pore protein FliP
		*motA*	Flagellar motor stator protein MotA
		*motB*	Flagellar motor protein MotB
		*ompA*	Porin OmpA
		*rcsC*	Two-component system sensor histidine kinase RcsC
		*rpoS*	RNA polymerase sigma factor RpoS
Secretion system	6	*rsmE*	16S rRNA (uracil (1498)-N(3))-methyltransferase
		*tssB*	Type VI secretion system contractile sheath small subunit
		*tssH*	Type VI secretion system ATPase TssH
		*tssJ*	Type VI secretion system lipoprotein TssJ
		*tssM*	Type VI secretion system membrane subunit TssM
		*vgrG*	Type VI secretion system tip protein VgrG
Toxin	2	*fabG*	3-oxoacyl-ACP reductase FabG
		*fabD*	ACP S-malonyltransferase

### Functional Validation of Potential Virulence-Related Genes in Strain Pa82

**FIGURE 6:**
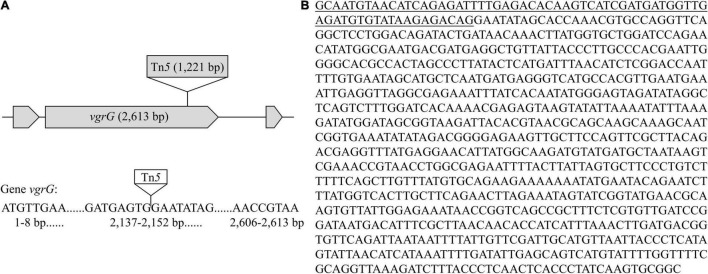
Characterization and sequence analysis of mutant Pa82-87-1. **(A)** Schematic diagram of Tn*5* transposon insertion in the *vgrG* gene; **(B)** Flanking sequence adjacent to Tn*5* transposon insertion site in mutant Pa82-87-1. The underlined part is the residual sequence of Tn*5* transposon.

**FIGURE 7:**
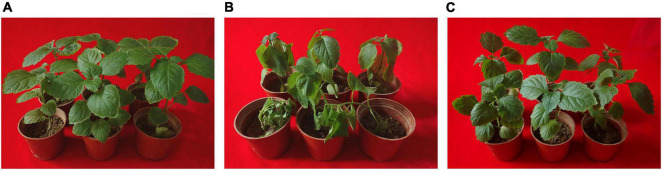
Pathogenicity test of mutant Pa82-87-1 on patchouli plants (at 5 days post-inoculation). **(A)** Plants mock-inoculated with sterile water (negative control); **(B)** Plants inoculated with wild type strain Pa82 (positive control); **(C)** Plants inoculated with mutant Pa82-87-1.

Functional validation of potential virulence-related genes in strain Pa82 requires construction of gene disruption mutants and identification of mutants defective in virulence. Mutants of strain Pa82 were created by random Tn*5* transposon mutagenesis and confirmed by PCR method. The flanking fragments adjacent to Tn*5* transposon insertion site in each mutant was individually amplified by inverse PCR, and amplified product was sequenced. The obtained sequences were compared for similarity to those available in GenBank database, and the potential virulence-related genes were selected. One such mutant (Pa82-87-1) was obtained, and the Tn*5* insertion junction was mapped to *vgrG*, a gene encoding one of type VI secretion system (T6SS) components (as shown in Table 3). Gene *vgrG* (2,613 bp in length) of mutant Pa82-87-1 was inserted by a 1,221 bp Tn*5* transposon (Figure 6). Pathogenicity test showed that mutant Pa82-87-1 was significantly reduced in virulence on patchouli plants compared to the wild type strain Pa82 (Figure 7). The results showed that *vgrG* gene in strain Pa82 was functionally validated as a virulence factor. In this way, more virulence-related genes of strain Pa82 may be further validated.

## Discussion

The genus *Kosakonia* has been recently derived from reclassification of genus *Enterobacter*, and several species, including *Enterobacter arachidis*, *Enterobacter cowanii*, *Enterobacter oryzae*, *Enterobacter radicincitans*, previously included in genus *Enterobacter* have been transferred to the novel genus *Kosakonia* (Brady et al., 2013). The genus *Kosakonia* has been reported to associate with plant growth-promoting bacteria (Kämpfer et al., 2005; Peng et al., 2009), and nowadays, some species from genus *Kosakonia* were found to be phytopathogen causing a variety of plant diseases (Furtado et al., 2012; Wu et al., 2016; Krawczyk and Borodynko-Filas, 2020). Bacterial wilt is one of the most serious threats to hundreds of plant species worldwide. For decades, the disease has been reported to be caused by *Ralstonia solanacearum*. However, several studies in recent years have indicated that some species from genus *Kosakonia* are capable of causing similar symptoms of bacterial wilt. For example, *Kosakonia cowanii* was identified as the causal agent of bacterial wilt on tomatoes (Sarkar and Chaudhuri, 2015). In this study, strain Pa82 was isolated from diseased patchouli plants with typical bacterial wilt symptoms and identified as *Kosakonia cowanii*. To our knowledge, this is the first report of *Kosakonia cowanii* Pa82 causing patchouli bacterial wilt in China.

Complete genome sequencing could provide novel insight into the genetic basis of virulence in strain Pa82. In this study, a total of 610 potential virulence-related genes were predicted in the genome of strain Pa82 through VFDB analysis. The roles of some genes in bacterial attachment, motility and virulence have been investigated in several animal and plant pathogenic bacteria. Gene *fimA* and gene *fimH* encoding adhesin mediating attachment of type 1 fimbriae are commonly associated with bacterial adhesion to host cells (Spaulding et al., 2017; Hasegawa and Nagano, 2021). Gene *flhC* and gene *flgK*, the regulating and structural flagellar genes, participate in the regulation of bacterial motility (Xu et al., 2014; Weller-Stuart et al., 2017). Gene *tssB* and gene *vgrG* related to the assembly of type VI secretion system (T6SS) have been reported to be important in the virulence of many animal and plant pathogens (Zhang et al., 2014; Wang et al., 2018). Detection of genes associated with virulence of strain Pa82 facilitates a better understanding how the bacterial pathogen has evolved virulence strategies to invade patchouli.

The genome of strain Pa82 has been completely sequenced and virulence-related genes have been predicted, the next step is often to validate roles of those genes in pathogenicity of the strain. The construction of gene disruption mutants generated by Tn*5* transposon insertion and their individual phenotype analysis is a common approach for the functional studies on the role of genes (Prelich, 2012). If Tn*5* transposon inserts into the virulence-related genes, the pathogenicity of the mutants may decrease or even disappear, which may identify these genes (Sakata et al., 2019). Our results showed that one such mutant with an insertion in *vgrG* gene (a gene encoding one of T6SS components) was obtained, it was therefore predicted to be affected in virulence phenotype. Pathogenicity test showed that the mutant was significantly reduced in virulence on patchouli plants compared to the wild type strain Pa82. In this study, an efficient method for functional validation of potential virulence-related genes in strain Pa82 was established.

## Data Availability Statement

The datasets presented in this study can be found in online repositories. The names of the repository/repositories and accession number(s) can be found below: https://www.ncbi.nlm.nih.gov/genbank/, CP069319, CP069320, CP069321, and CP069322.

## Author Contributions

HH was the principal investigator, conceived, and designed the experiments, and contributed to the writing and revision of the manuscript. YZ, BW, and QL performed the experiments, analyzed the data, and wrote the manuscript. DH wrote parts of the manuscript. YYZ and GL performed parts of the experiments. All authors approved the final version of the manuscript.

## Conflict of Interest

The authors declare that the research was conducted in the absence of any commercial or financial relationships that could be construed as a potential conflict of interest.

## Publisher’s Note

All claims expressed in this article are solely those of the authors and do not necessarily represent those of their affiliated organizations, or those of the publisher, the editors and the reviewers. Any product that may be evaluated in this article, or claim that may be made by its manufacturer, is not guaranteed or endorsed by the publisher.
